# Comprehensive analysis of the diatom genus *Psammodictyon* from Viet Nam: new species, molecular data, and fatty acid content

**DOI:** 10.3389/fmicb.2025.1701605

**Published:** 2025-12-12

**Authors:** Elena Kezlya, Dmitry Kapustin, Zinaida Krivova, Yevhen Maltsev, Lam Nguyen-Ngoc, Duyen Thi Ngoc Huynh, Hai Doan-Nhu, Maxim Kulikovskiy

**Affiliations:** 1 K. A. Timiryazev Institute of Plant Physiology, Russian Academy of Sciences, Moscow, Russia; 2 Institute of Oceanography, Viet Nam Academy of Science and Technology, Nha Trang, Viet Nam

**Keywords:** diatoms, morphology, taxonomy, phylogeny, SEM, PUFA

## Abstract

**Introduction:**

*Psammodictyon* is a widely distributed marine diatom genus with a complex taxonomy and underestimated species diversity.

**Methods:**

Light and scanning electron microscopy, phylogenetic analysis and gas chromatography were used for study of the newly isolated strains of *Psammodictyon*.

**Results:**

Twelve strains of *Psammodictyon* were isolated from the coastal waters of central Viet Nam. Based on detailed morphological examinations using light and scanning electron microscopy, as well as molecular phylogenetic analysis, we propose six new species to science. Our phylogenetic analysis indicates that *Psammodictyon* forms a closely related monophyletic group. The resolution power of the short genetic markers (V4, V9 18S rRNA, V9-ITS1 18S rRNA, and a short region of *rbcL*), which are widely used for metabarcoding, is also discussed. Analysis of the fatty acid content of seven *Psammodictyon* strains showed that, like most diatoms, they accumulate high levels of polyunsaturated fatty acids (29.44–42.62%) and monounsaturated fatty acids (20.2–23.2%).

**Conclusion:**

The number of known species in the genus *Psammodictyon* has increased to 21. *Psammodictyon* strains may be considered as potential producers of long-chain omega-3 polyunsaturated fatty acids.

## 1 Introduction

*Psammodictyon* is a mainly marine genus of canal-raphe-bearing diatoms belonging to the family Bacillariaceae. It was described by Mann (Round et al., 1990) with *Psammodictyon panduriforme* (W. Gregory) D. G. Mann (≡*Nitzschia panduriformis* W. Gregory) as its type. In contrast to *Nitzschia*, the valves of *Psammodictyon* are panduriform with an undulate surface and the areolae are loculate (chambered), creating decussate (transapical and diagonally orientated) striae (Round et al., 1990; Kezlya et al., 2025b).

*Psammodictyon* taxa are often common and/or dominant in various biotopes and geographic regions, with *Psammodictyon panduriforme* and *P. constrictum* (W. Gregory) D. G. Mann being mentioned most frequently. Both taxa were described by Gregory as *Nitzschia panduriformis* Gregory (1857) and *Tryblionella constricta* Gregory (1855), respectively. The latter species was reclassified within *Nitzschia* (Cleve and Grunow, 1880). The incompleteness of Gregory’s descriptions led to the misapplication of these names. The taxa described and illustrated as *Nitzschia panduriformis* and *N. constricta* in identification texts (e.g., Van Heurck, 1881, 1885; Peragallo and Peragallo 1897–1908; Witkowski et al., 2000) actually represent other species. Recently, Mann and Trobajo (2025) noted a similar problem for *Tryblionella compressa* (Bailey) Poulin. Witkowski et al. (2000) emphasized that the entire complex around *Nitzschia panduriformis* requires a general taxonomic revision. A study of the type specimens of both *Psammodictyon panduriforme* and *P. constrictum* will help to better understand their identity and actual geographic distribution.

The favorable growth characteristics of *Psammodictyon* taxa have allowed their strains to be used in applied research. For instance, Camargo et al. (2016) studied optical and material properties of the *P. panduriforme* frustules and showed that they have quasi-regular pore patterns on the biosilica valves, which can be utilized in optoelectronic devices with mesoporous structures. Guan et al. (2019) investigated two strains of canal-raphe-bearing diatoms, *Nitzschia bilobata* (AQ1) and *Psammodictyon panduriforme* (NP), as cation exchange materials for lysozyme purification from chicken egg white. They demonstrated that diatom frustules are more effective and could serve as an alternative chromatographic matrix for lysozyme purification.

It should be noted that the strains used in these studies obviously do not belong to *Psammodictyon panduriforme*, as their valve shape and size do not correspond with those reported by Gregory (1857). Correct species identification is crucial for applied research.

Lipids are the significant components of diatom cells. The lipid content in diatom algae can reach up to 25% of dry weight, although the production of lipids depends on growth conditions (Yi et al., 2017). Diatom lipids comprise a diverse group of fatty acids, including saturated fatty acids (SFAs), monounsaturated fatty acids (MUFAs), and polyunsaturated fatty acids (PUFAs) (Pekkoh et al., 2022). Omega-3 fatty acids are essential for human health and wellness, particularly eicosapentaenoic acid (EPA) and docosahexaenoic acid (DHA), two long-chain polyunsaturated fatty acids (Tyagi et al., 2024). Despite significant progress in fatty acids research, there has been only one investigation on the lipid content of two *Psammodictyon* strains (Krishnaswami et al., 2024).

The phylogenetic placement of the genus *Psammodictyon* has been repeatedly shown in previous studies. Representatives of *Psammodictyon* form a well-supported clade within the Bacillariales, demonstrating monophyly (Rimet et al., 2011; Witkowski et al., 2015; Ashworth et al., 2017; Carballeira et al., 2017; Sabir et al., 2018; Mann et al., 2021; Mucko et al., 2021; Olszyński et al., 2025). However, in these studies, no more than six strains were included in the analysis, and a detailed assessment of phylogenetic relationships within the *Psammodictyon* was not performed.

This study aims to examine the morphology, molecular phylogeny (based on 18S rRNA, *rbc*L, and internal transcribed spacer regions), and fatty acid content of *Psammodictyon* strains isolated from the coastal waters of Viet Nam.

## 2 Materials and methods

### 2.1 Sampling

**Figure 1:**
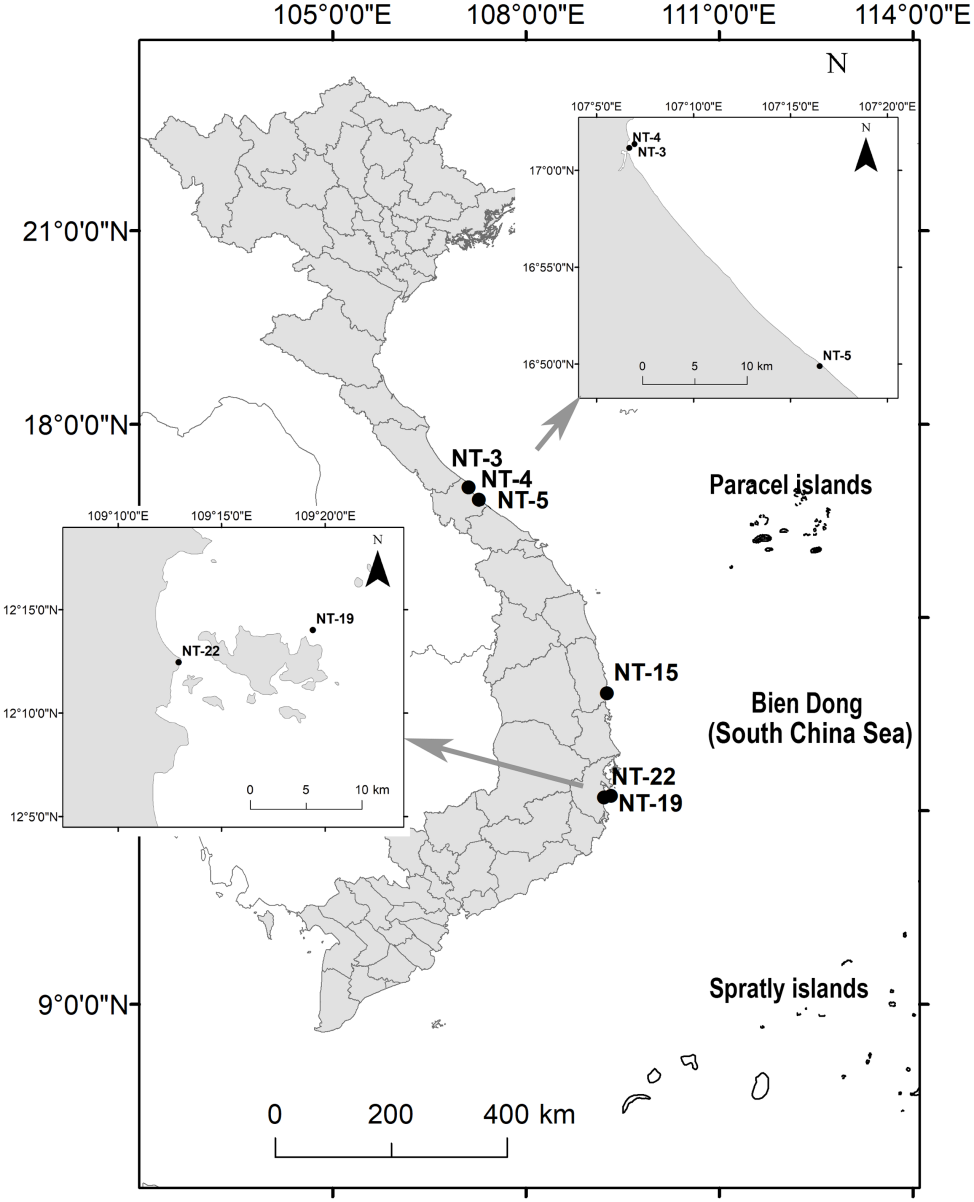
Location of the sampling sites.

The samples used in this study were collected along the coast of central Viet Nam (Figure 1). Both phytoplankton and epiphyte samples were collected. For phytoplankton samples, the surface water was filtered through a 29-μm plankton net and transferred into 50 mL tubes. For epiphytes samples, the macrophytes were scraped into a tray with approximately 50 mL of seawater, and the resulting suspension was transferred into 15 mL tubes. All samples were stored at room temperature until further analysis in the laboratory.

Water salinity and temperature were measured using an Atago PAL-06S seawater refractometer (Atago, Japan).

### 2.2 Culturing

A subsample was added to the enriched seawater, artificial water liquid medium (Andersen and Kawachi, 2005; Polyakova et al., 2018). A monoclonal strain was established by micropipetting a single cell under a Zeiss Axio Vert. A1 inverted microscope. A non-axenic monoculture was cultivated in ESAW liquid medium in Petri dishes at 22–25 °C with an alternating 12-h light and dark photoperiod. The strain was analyzed after 1 month of culturing. A list of all strains examined in this study and the geographic locations of sampling sites with measured ecological parameters is presented in Table 1.

**Table 1:** List of the sampling sites with environmental variables and studied strains.

Sampling date	Locality, coordinates	Sample number, biotope	Salinity (‰)	Temperature °C	Species	Strain	Slide number
23.07.2024	Cua Tung, Quang Tri N 17.022566, E 107.116105	NT-3 (5), plankton	34	29	*Psammodictyon haii* sp. nov.	CBMCsvn745	HD 09813
					*Psammodictyon similis* sp. nov.	CBMCsvn749	HD 09817
						CBMCsvn750	HD 09818
23.07.2024	Cua Tung, Quang Tri, N 17.019236, E 107.111506	NT-4 (9), epizoic on mollusk shell	33	29	*Psammodictyon minutum* sp. nov.	CBMCsvn943	HD 10026
24.07.2024	Trieu Co, Quang Tri N 16.831604; E 107.275227	NT-5 (10), plankton	32	28.3	*Psammodictyon lamii* sp. nov.	CBMCsvn758	HD 09826
27.07.2024	Thi Nai lagoon, Qui Nhon, N 13.827088, E 109.258570	NT-15 (40), plankton	34	28.0	*Psammodictyon pusillum* sp. nov.	CBMCsvn861	HD 09945
					*Psammodictyon lanceolatum* sp. nov.	CBMCsvn866	HD 09950
30.07.2024	Nha Trang, Hon Tre island, N 12.233617, E 109.323633	NT-19 (58), plankton	34	28.3	*Psammodictyon* cf. *constrictum*	CBMCsvn771	HD 09839
						CBMCsvn773	HD 09841
	Nha Trang, N 12.2075779, E 109.2155615	NT-22 (66), epiphytes on *Sargassum* sp.	34	28.3	*Psammodictyon minutum* sp. nov.	CBMCsvn846	HD 09931
					*Psammodictyon pusillum* sp. nov.	CBMCsvn835	HD 09920
						CBMCsvn839	HD 09924
20.04.2018	Nha Trang, N 12.207577778, E 109.215561389	NT-22 (2018), Epilithon			*Psammodictyon crassum*	CBMCsvn634	HD 09251

### 2.3 Preparation of slides and microscope investigation

Strains for light microscopic (LM) and scanning electron microscopy investigations were processed following a standard procedure involving treatment with concentrated hydrogen peroxide and final washes with distilled water (Kezlya et al., 2020). Permanent diatom preparations were mounted in Naphrax^®^ (Brunel Microscopes Ltd., Chippenham, United Kingdom; refractive index = 1.73). LM observations were performed using an AxioScope A1 microscope (Zeiss, Germany) equipped with an oil immersion objective (×100/n.a.1.4, differential interference contrast) and an AxioCam Erc 5s camera in the Laboratory of Molecular Systematics of Aquatic Plants, K. A. Timiryazev Institute of Plant Physiology, Russian Academy of Sciences. The ultrastructure of the valves was examined with a TESCAN Vega III scanning electron microscope (TESCAN, Brno, Czech Republic) in the Borissiak Paleontological Institute, Russian Academy of Sciences.

### 2.4 Molecular study

Total DNA from the studied strain was extracted using Chelex 100 Chelating Resin (Bio-Rad Laboratories, Hercules, CA, United States) according to protocol 2.2. Nuclear gene V4 and V9-ITS1 regions of 18S rRNA and plastid *rbc*L gene were amplified. For the highly variable V4 region of 18S rRNA (~390 bp), primers D512for and D978rev were used (Zimmermann et al., 2011). For the highly variable V9-ITS1 region of 18S rRNA (~430–580 bp), primers Euk1391F and ITS2_broad (Boenigk et al., 2018) were used. The plastid *rbc*L was amplified using two pairs of primers *rbc*L40 + and *rbc*L587-, and *rbc*L404 + and *rbc*L1444 (Ruck and Theriot, 2011). Polymerase chain reaction amplifications were performed using premade mastermixes (ScreenMix, Evrogen, Moscow, Russia).

Amplification of the V4 region of 18S rDNA was performed using the following program: initial denaturation at 95 °C for 5 min; followed by 35 cycles of denaturation at 94 °C for 30 s, annealing at 52 °C for 30 s, and elongation at 72 °C for 50 s; and a final extension at 72 °C for 7 min, then held at 12 °C. Amplification of the V9-ITS1 region of 18S rDNA region was performed using the following program: initial denaturation of 5 min at 96 °C; followed by 35 cycles of denaturation at 96 °C (30 s), annealing at 52 °C (60 s), and elongation at 72 °C (75 s); and a final extension at 72 °C (10 min), then held at 12 °C. Amplification of the *rbc*L gene was performed using the following program: initial denaturation of 4 min at 94 °C; followed by 44 cycles of denaturation at 94 °C (50 s), annealing at 53 °C (50 s), and elongation at 72 °C (80 s); and a final extension at 72 °C (10 min), then held at 12 °C.

The PCR products were visualized on a 1.0% agarose gel stained with SYBR™ Safe (Life Technologies, Carlsbad, CA, United States) and then purified using a mixture of FastAP, 10 × FastAP Buffer, Exonuclease I (Thermo Fisher Scientific, Waltham, MA, United States), and water. The purified PCR products were sequenced using the Sanger Sequencing method by “Syntol” scientific production company (Moscow).

Newly obtained sequences were manually edited in Ridom TraceEdit ver. 1.1.0 (Ridom GmbH, Münster, Germany) and Mega ver. 11 (Kumar et al., 2016). For two-gene analysis (V4 18S rRNA and *rbc*L), the reads were combined with GenBank-extracted sequences of 69 diatom strains from the family Bacillariaceae, including *Nitzschia* Hassall, *Tryblionella* W. Smith, *Bacillaria* J. F. Gmelin, *Cylindrotheca* Rabenhorst, *Denticula* Kützing, and *Psammodictyon*. Four *Actinocyclus* Ehrenberg strains were chosen as an outgroup (taxon names and accession numbers are provided in Supplementary Table S1). The 18S rDNA and *rbc*L sequences were aligned separately using the G-INS-i algorithm in the Mafft ver. 7 (RIMD, Osaka, Japan) (Katoh and Toh, 2010). The resulting dataset comprised 385 nucleotide sites of nuclear 18S rDNA and 1,229 sites of plastid *rbc*L regions. After removal of unpaired regions, the aligned 18S rRNA gene sequences were combined with the *rbc*L gene sequences into a single matrix for a concatenated *rbc*L and 18S rDNA tree.

The Bayesian inference (BI) method was performed to infer the phylogenetic position of new diatom strains using Beast ver. 1.10.1 (BEAST Developers, Auck-land, New Zealand) (Drummond and Rambaut, 2007). The most appropriate partition-specific substitution models, shape parameters *α*, and a proportion of invariable sites (pinvar) were determined by the Bayesian information criterion (BIC) in jModel-Test ver. 2.1.10 (Vigo, Spain) (Darriba et al., 2012). This BIC-based model selection procedure selected the following models, shape parameter (α), and proportions of invariable sites (pinvar): HKY + I + G, α = 0.3410 and pinvar = 0.4670 for 18S rDNA; HKY + I + G, α = 0.4390 and pinvar = 0.6840 for the first codon position of the *rbc*L gene; JC + I, pinvar = 0.8850 for the second codon position of the *rbc*L gene; GTR + I + G, α = 0.8890 and pinvar = 0.1750 for the third codon position of the *rbc*L gene. A speciation model was performed by a Yule process tree prior. Five Markov chain Monte Carlo analyses were run for seven million generations. The convergence diagnostics were performed in Tracer ver. 1.7.1 (MCMC Trace Analysis Tool, Edinburgh, United Kingdom) (Drummond and Rambaut, 2007). The initial 10% of trees were removed, and the remaining trees were used to construct a final chronogram with 90% posterior probabilities. Trees were viewed and edited using FigTree ver. 1.4.4 (University of Edinburgh, Edinburgh, United Kingdom) and Adobe Photoshop CC ver. 19.0.

The V4 and V9, V9-ITS1 regions of 18S rDNA and *rbc*L sequences were also used to estimate the degree of similarity between gene sequences of different *Psammodictyon* strains (Supplementary Tables S2–S7). Using the Mega11, p-distances were determined to calculate sequence similarity using the formula (1–p) × 100. *Tryblionella apiculata* TRY946CAT was included in the analysis to estimate intergeneric genetic distances.

### 2.5 Fatty acid analysis

Biomass preparation for determining the fatty acid methyl ester (FAME) profiles was performed according to Maltsev et al. (2021). The diatom suspensions were conveyed to 15–50 mL tubes (depending on the volume). The cells were pelleted at room temperature for 3 min at 3600 g (Cence CHT210R (China)). The supernatant was removed, and the pelleted cells were resuspended in 10–15 mL (depending on the amount of biomass) of distilled water, quantitatively transferred to 15 mL centrifuge tubes, and pelleted again by centrifugation. The supernatant was removed, and the samples were quantitatively transferred to a 50-ml round-bottom flask. Heptadecanoic acid (Sigma-Aldrich, St. Louis, MO, United States) was used as the internal standard for the fatty acid composition determination. To avoid the oxidation of unsaturated fatty acids, all samples were processed under an argon atmosphere. Ten milliliters of 1 M KOH in 80% aqueous ethanol was added to the dry residue, the flask was sealed with a reflux condenser, and the mixture was maintained for 60 min at the boiling point (~80 °С). After this, the solvents were evaporated *in vacuo* to a volume of ~3 mL and quantitatively transferred with distilled water to a 50-ml centrifuge tube to a total volume of 25 mL, followed by extraction of unsaponifiable components with 10-ml portions of n-hexane (Himmed, Moscow, Russia) three times. To accelerate phase separation, the tube was centrifuged for 5 min at room temperature and at 2022xg. The aqueous phase was then acidified to a slightly acidic reaction (as indicated by indicator paper) with a few drops of 20% sulfuric acid (Himmed, Moscow, Russia), and free fatty acids were extracted with 20 mL of n-hexane. The n-hexane solution of free fatty acids was transferred to a dry 50-ml round-bottom flask, and the solvent was evaporated to dryness using a rotary evaporator IKA RV-10 (IKA-WERKE, Staufen im Breisgau, Germany), after which 10 mL of absolute methanol (Sigma-Aldrich, St. Louis, MO, United States) and 1 mL of acetyl chloride (Sigma-Aldrich, St. Louis, MO, United States) were added to the dry residue. The flask, closed with a reflux condenser, was maintained for 1 h at 70 °С. The solvents were evaporated to dryness, a few drops of distilled water were added to the dry residue, and FAMEs were extracted with n-hexane.

The obtained FAMEs were analyzed using a Chromatec Crystal 5000.2 NP device (ZAO SKB Chromatec, Russian Federation) equipped with a quadrupole mass spectrometric detector. A Restek Rtx-2330 capillary column (60 m; cat. no. 10726 Restek, United States) was used. The operating conditions were as follows: carrier gas linear velocity, 1 mL/min; injection volume, 1 μL; split ratio, 1:20; evaporator temperature, 260°С. The temperature program for gradient analysis was as follows: a plateau at 60°С for 8 min, heating to 170°С at a rate of 10°С/min and maintaining this temperature for 5 min, followed by heating from 170°С to 245°С at a rate of 6.5°С/min and maintaining this temperature until the end of the analysis. The detector temperature was 230°С, and the ionization energy was 70 eV. Identification and quantification of FAMEs were performed using Chromatec Analyst 3 software. All experiments and analyses were conducted in triplicate. Mean values and standard errors of the mean are presented in Table 2.

**Table 2:** Fatty acid composition of new *Psammodictyon* strains (% of total fatty acids).

Fatty acid	*P. haii* sp. nov. CBMCsvn745	*P. similis* sp. nov. CBMCsvn749	*P. similis* sp. nov. CBMCsvn750	*P. lamii* sp. nov. CBMCsvn758	*P.* cf. *constrictum* CBMCsvn773	*P. pussilum* sp. nov. CBMCsvn839	*P. minutum* sp. nov. CBMCsvn846
16:00 Palmitic acid	35.08 ± 0.1	35.03 ± 0.3	37.94 ± 0.4	38 ± 0.5	37.56 ± 0.4	34.83 ± 0.6	22.07 ± 0.7
18:00 Stearic acid	9.78 ± 0.2	5.66 ± 0.5	7.43 ± 0.6	10.07 ± 0.1	10.6 ± 0.3	10.41 ± 0.2	10.78 ± 0.3
22:00 Begenic acid	−	1.82 ± 0.3	3.01 ± 0.2	−	−	−	1.33 ± 0.5
16:1n-7 cis-9-Palmitoleic acid	20.65 ± 0.2	20.2 ± 0.5	21.56 ± 0.4	22.9 ± 0.5	21.92 ± 0.2	21.39 ± 0.5	23.2 ± 0.5
22:4(n-6) cis-7,10,13,16-Docosatetraenoic acid/Adrenic acid	−	7.94 ± 0.3	−	2.12 ± 0.2	−	6.15 ± 0.4	6.5 ± 0.4
20:5(n − 3) cis-5,8,11,14,17-Eicosapentaenoic acid	12.1 ± 0.2	15.82 ± 0.7	13.56 ± 0.7	14.75 ± 0.5	13.69 ± 0.6	12.74 ± 0.1	13.43 ± 0.4
22:6(n-3) cis-4,7,10,13,16,19-Docosahexaenoic acid	22.39 ± 0.2	13.53 ± 0.4	16.5 ± 0.7	12.57 ± 0.4	16.23 ± 0.5	14.48 ± 0.7	22.69 ± 0.7
Total SFAs	44.86	42.51	48.38	48.07	48.16	45.24	34.18
Total MUFAs	20.65	20.2	21.56	22.49	21.92	21.39	23.2
Total PUFAs	34.49	37.29	30.06	29.44	29.92	33.37	42.62

## 3 Results

### 3.1 Taxonomy

**Figure 2:**
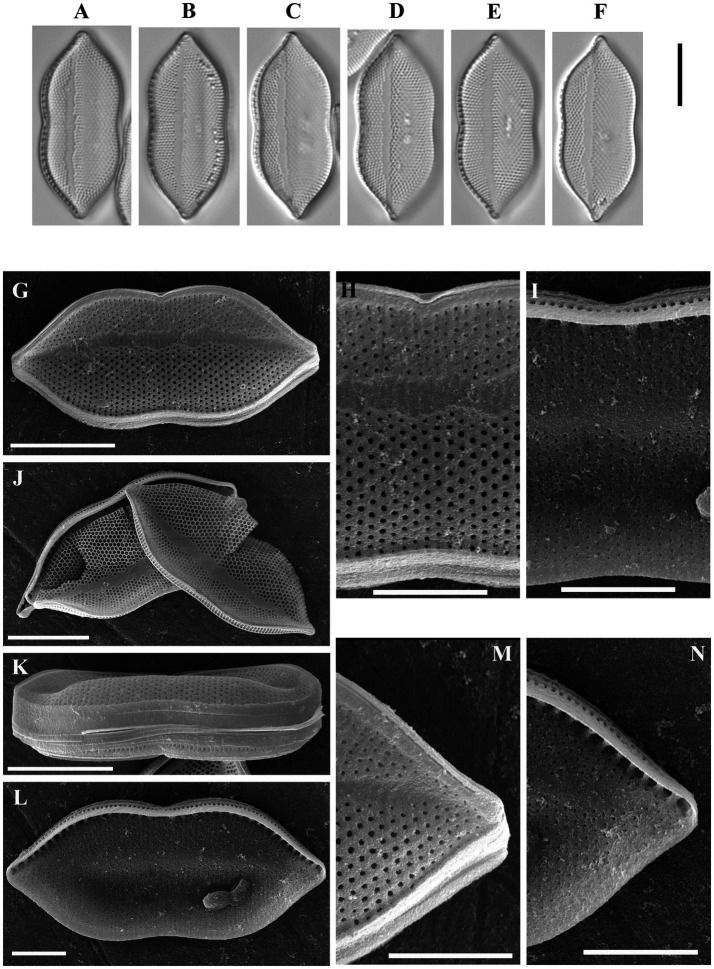
*Psammodictyon lamii* sp. nov. **(A−F)** Size diminution series, LM; **(G)** valve external view, SEM; **(H)** close-up view of the central part externally, SEM; **(I)** close-up view of the central part internally, SEM; **(J)** two valves showing hexagonal structure of the locular areolae, SEM; **(K)** girdle view (note the elevated distal part of the valve), SEM; **(L)** valve internal view, SEM; **(M)** close-up view of the apices externally, SEM; **(N)** close-up view of the apices internally, SEM. Scale bars: **(A–G,J,K)** = 10 μm; **(H,I,L–N)** = 5 μm.

*Psammodictyon lamii* Kapustin, Kezlya and Kulikovskiy sp. nov. (Figure 2).

Description: LM: Valves broadly lanceolate, slightly constricted in the middle, with subrostrate ends, a longitudinal fold along the apical axis and a narrow hyaline area shifted to the valve margin, 29.5–30 μm long, 11.5–13 μm broad in the widest part, and 11–12 μm at the constriction. Keeled raphe system present on valve margin, with 9–10 distinct fibulae in 10 μm, the middle pair more distant from others. Striae 20 in 10 μm, interrupted by a narrow hyaline area, areolae arranged in quincunx. SEM: Externally, the proximal valve side (i.e., the part next to the raphe) depressed and separated from the elevated distal side by a hyaline sternum. Striae decussate, composed of loculate areolae. External areolar openings round. External raphe fissures accompanied by a ridge. Central raphe endings separated by a nodule. Distal raphe endings deflected to the proximal valve side (see Figure 2M). Internally loculate areolae composed of hexagonal chambers (see Figure 2J). Inner areolae openings round, slightly depressed (see Figures 2L,I,N). Raphe terminates at apices in small helictoglossae.

Holotype: Permanent slide HD 09826, deposited at the K. A. Timiryazev Institute of Plant Physiology, Russian Academy of Sciences (HD), prepared from oxidized culture strain CBMCsvn758 isolated from sample NT-5 (10). Holotype illustrated in Figure 2A.

Isotype: Permanent slide 09826a, deposited at the Oceanographic Museum, Institute of Oceanography, Nha Trang, Viet Nam, with the accession number VMO: E 58216. Herbarium registration code VMO.

Type locality: Viet Nam, South China Sea, Trieu Co, Quang Tri, plankton (N 16.831604, E 107.275227), *leg.* E. Kezlya and D. Kapustin, 24 July 2024.

Reference strain. CBMCsvn758 deposited at the Culture and Barcode Collection of Microalgae and Cyanobacteria “AlgaBank” (CBMC), K. A. Timiryazwev Institute of Plant Physiology, Russian Academy of Sciences and at the Microalgal Culture Collection of Institute of Oceanography, Viet Nam.

Sequence data: Partial 18S rRNA gene sequence comprising V4 domain sequence (GenBank accession number PX596449), partial 18S rRNA gene sequence comprising V9-ITS1 domain sequence (GenBank accession number PX560120), and partial *rbcL* sequence (GenBank accession number PX607289) for the strain CBMCsvn758.

Registration: https://phycobank.org/106048.

Etymology: The species is named after Prof. Lam Nguyen-Ngoc (Institute of Oceanography, Viet Nam) for his contribution to the study of algae in Viet Nam.

**Figure 3:**
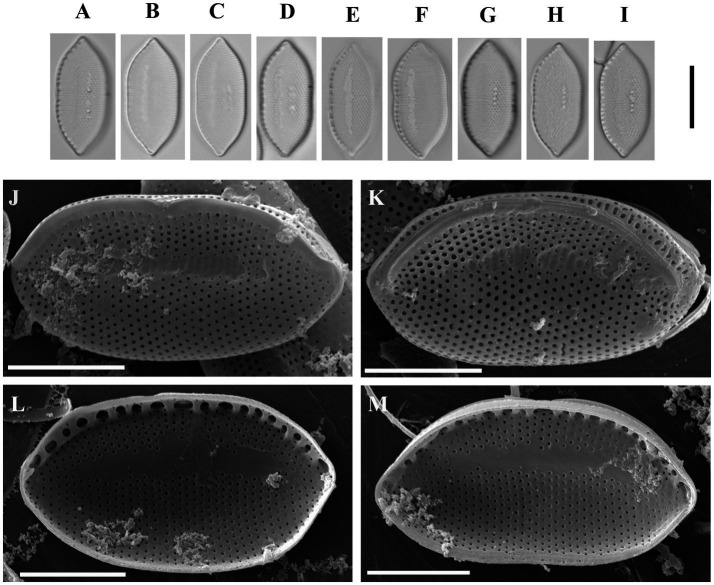
*Psammodictyon haii* sp. nov. **(A−I)** Size diminution series, LM; **(J,K)** valve external view, SEM; **(L,M)** valve internal view, SEM. Scale bars: **(A−I)** = 10 μm; **(J−M)** = 5 μm.

Psammodictyon haii Kapustin, Kezlya and Kulikovskiy sp. nov. (Figure 3).

Description: LM: Valves elliptic with almost parallel margins, ends cuneate to subrostrate, 17.5–19 μm long and 7.5–8.5 μm broad. Keeled raphe system present on valve margin, with 11–13 distinct fibulae in 10 μm, the middle pair more distant from others. Striae 27–29 in 10 μm, interrupted by a narrow hyaline area, areolae arranged in quincunx. SEM: Externally the proximal valve side (i.e., the part next to the raphe) slightly depressed and separated from the elevated distal side by a hyaline sternum. Striae decussate, composed of loculate areolae. External areolar openings round. External raphe fissures accompanied by a ridge. Central raphe endings separated by a nodule. Distal raphe endings hooked (see Figure 3K). Inner areolae openings round, slightly depressed. Raphe terminates at apices in small helictoglossae.

Holotype: Permanent slide HD 09813, deposited at the K. A. Timiryazev Institute of Plant Physiology, Russian Academy of Sciences (HD) prepared from oxidized culture strain CBMCsvn745 isolated from sample NT-3 (5). Holotype illustrated in Figure 3F.

Isotype: Permanent slide 09813a, deposited at the Oceanographic Museum, Institute of Oceanography, Nha Trang, Viet Nam, with the accession number VMO: E 58217. Herbarium registration code VMO.

Type locality: Viet Nam, South China Sea, Cua Tung, Quang Tri, plankton (N 17.022566, E 107.116105), *leg.* E. Kezlya and D. Kapustin, 23 July 2024.

Reference strain: CBMCsvn745 deposited at the Culture and Barcode Collection of Microalgae and Cyanobacteria “AlgaBank” (CBMC), K. A. Timiryazev Institute of Plant Physiology, Russian Academy of Sciences and at the Microalgal Culture Collection of Institute of Oceanography, Viet Nam.

Sequence data: Partial 18S rRNA gene sequence comprising V4 domain sequence (GenBank accession number PX596448), partial 18S rRNA gene sequence comprising V9-ITS1 domain sequence (GenBank accession number PX560122), and partial *rbcL* sequence (GenBank accession number PX607290) for the strain CBMCsvn745.

Registration: https://phycobank.org/106049.

Etymology: The species is named after Dr. Hai Doan-Nhu (Institute of Oceanography, Viet Nam) for his contribution to the study of algae in Viet Nam.

**Figure 4:**
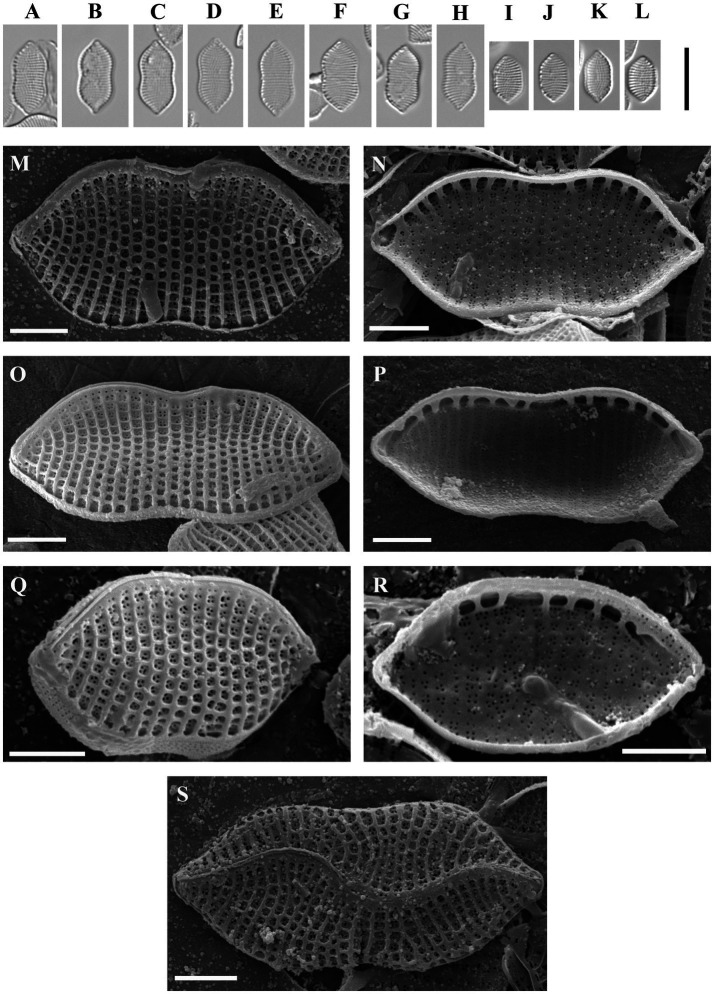
*Psammodictyon pusillum* sp. nov. **(A−L)** Size diminution series, LM; **(A−C)** strain CBMCsvn835; **(D−H)** strain CBMCsvn839; **(I−L)** strain CBMCsvn861; **(M)** valve external view, strain CBMCsvn839, SEM; **(N)** valve internal view, strain CBMCsvn839, SEM; **(O)** valve external view, strain CBMCsvn835, SEM; **(P)** valve internal view, strain CBMCsvn835, SEM; **(Q)** valve external view, strain CBMCsvn861, SEM; **(R)** valve internal view, strain CBMCsvn861, SEM; **(S)** teratological form, strain CBMCsvn839. Note the presence of the undulate raphe on the valve surface. Scale bars: **(A–L)** = 10 μm; **(M−S)** = 2 μm.

Psammodictyon pusillum Kapustin, Kezlya and Kulikovskiy sp. nov. (Figure 4).

Description: LM: Valves panduriform, strongly constricted in the middle, with cuneate to subrostrate ends, 7–12 μm long, 4.5–6 μm broad in the widest part, and 4.5–5 μm at the constriction. Keeled raphe system present on valve margin, with 16–19 distinct fibulae in 10 μm. Striae 22–24 in 10 μm. SEM: Striae composed of loculate areolae with rectangular chambers. External raphe fissures accompanied by a ridge. Central raphe endings separated by a nodule. Distal raphe endings deflected to the mantle. Internally areolae have (2)3–4(6) openings. Raphe terminates at apices in small helictoglossae.

Holotype: Permanent slide HD 09924, deposited at the K. A. Timiryazev Institute of Plant Physiology, Russian Academy of Sciences (HD), prepared from oxidized culture strain CBMCsvn839 isolated from sample NT-22 (66). Holotype illustrated in Figure 4A.

Isotype: Permanent slide 09924a, deposited at the Oceanographic Museum, Institute of Oceanography, Nha Trang, Viet Nam, with the accession number VMO: E 58218. Herbarium registration code VMO.

Type locality: Viet Nam, South China Sea, Nha Trang Bay, epiphytes on *Sargassum* sp. (N 12.2075779, E 109.2155615), *leg.* E. Kezlya and D. Kapustin, 30 July 2024.

Reference strain: CBMCsvn839 deposited at the Culture and Barcode Collection of Microalgae and Cyanobacteria “AlgaBank” (CBMC), K. A. Timiryazev Institute of Plant Physiology, Russian Academy of Sciences and at the Microalgal Culture Collection of Institute of Oceanography, Viet Nam.

Sequence data: Partial 18S rRNA gene sequence comprising V4 domain sequence (GenBank accession number PX596451), partial 18S rRNA gene sequence comprising V9-ITS1 domain sequence (GenBank accession number PX560121) and partial *rbcL* sequence (GenBank accession number PX607291) for the strain CBMCsvn839. Partial 18S rRNA gene sequence comprising V4 domain sequence (GenBank accession number PX596450), partial 18S rRNA gene sequence comprising V9-ITS1 domain sequence (GenBank accession number PX560123) and partial *rbcL* sequence (GenBank accession number PX607292) for the strain CBMCsvn835. Partial 18S rRNA gene sequence comprising V4 domain sequence (GenBank accession number PX596458), partial 18S rRNA gene sequence comprising V9-ITS1 domain sequence (GenBank accession number PX560125) and partial *rbcL* sequence (GenBank accession number PX607293) for the strain CBMCsvn861.

Representative specimens. Strain CBMCsvn835 (slide no. 09920, sample no. NT-22 (66) epiphytes on *Sargassum* sp.), strain CBMCsvn861 (slide no. 09945, sample no. NT-15 (40),) plankton).

Registration: https://phycobank.org/106050.

Etymology: The species epithet reflects the small size of this species (Latin *“pusillum”* means *“little”*).

**Figure 5:**
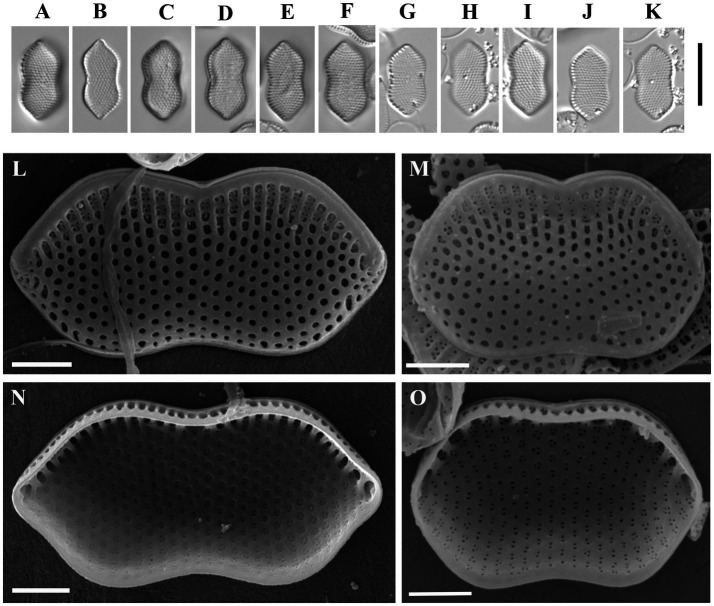
*Psammodictyon minutum* sp. nov. **(A−K)** Size diminution series, LM; **(A−F)** strain CBMCsvn943; **(G−K)** strain CBMCsvn846; **(L)** valve external view, strain CBMCsvn943, SEM; **(M)** valve external view, strain CBMCsvn846, SEM; **(N)** valve internal view, strain CBMCsvn943, SEM; **(O)** valve internal view, strain CBMCsvn846, SEM. Scale bars: **(A–K)** = 10 μm; **(L−O)** = 2 μm.

Psammodictyon minutum Kapustin, Kezlya and Kulikovskiy sp. nov. (Figure 5).

Description: LM: Valves panduriform, strongly constricted in the middle, with cuneate to subrostrate ends, 11–13.5 μm long, 5.5–6.5 μm broad in the widest part, and 4.5–5.5 μm at the constriction. Keeled raphe system present on valve margin, with 16 fibulae in 10 μm. Striae 22 in 10 μm. SEM: Striae composed of loculate areolae with rectangular chambers. External locular openings round, except for those close to the proximal valve margin. External raphe fissures accompanied by a ridge. Central raphe endings separated by a nodule. Distal raphe endings deflected to the mantle. Girdle bands open. Internally areolae have (2)3–4 openings. Raphe terminates at apices in small helictoglossae.

Holotype: Permanent slide HD 10026, deposited at the K. A. Timiryazev Institute of Plant Physiology, Russian Academy of Sciences (HD), prepared from oxidized culture strain CBMCsvn943 isolated from sample NT-4 (9). Holotype illustrated in Figure 5E.

Isotype: Permanent slide 10026a, deposited at the Oceanographic Museum, Institute of Oceanography, Nha Trang, Viet Nam, with the accession number VMO: E 58219. Herbarium registration code VMO.

Type locality: Viet Nam, South China Sea, Cua Tung, Quang Tri, epizoic on mollusc shell (N 17.019236, E 107.111506), *leg*. E. Kezlya and D. Kapustin, 23 July 2024.

Reference strain: CBMCsvn943 deposited at the Culture and Barcode Collection of Microalgae and Cyanobacteria “AlgaBank” (CBMC), K. A. Timiryazev Institute of Plant Physiology, Russian Academy of Sciences and at the Microalgal Culture Collection of Institute of Oceanography, Viet Nam.

Representative specimen. Strain CBMCsvn846 (slide no. 09931, sample no. NT-22 (66), epiphytes on *Sargassum* sp).

Sequence data: Partial 18S rRNA gene sequence comprising V4 domain sequence (GenBank accession number PX596452) partial 18S rRNA gene sequence comprising V9-ITS1 domain sequence (GenBank accession number PX560124) and partial *rbcL* sequence (GenBank accession number PX607294) for the strain CBMCsvn943. Partial 18S rRNA gene sequence comprising V4 domain sequence (GenBank accession number PX596453), partial 18S rRNA gene sequence comprising V9-ITS1 domain sequence (GenBank accession number PX560126) and partial *rbcL* sequence (GenBank accession number PX607295) for the strain CBMCsvn846.

Registration: https://phycobank.org/106051.

Etymology: The species epithet reflects the small size of this species (Latin *“minutum”* means *“minute, small”*).

**Figure 6:**
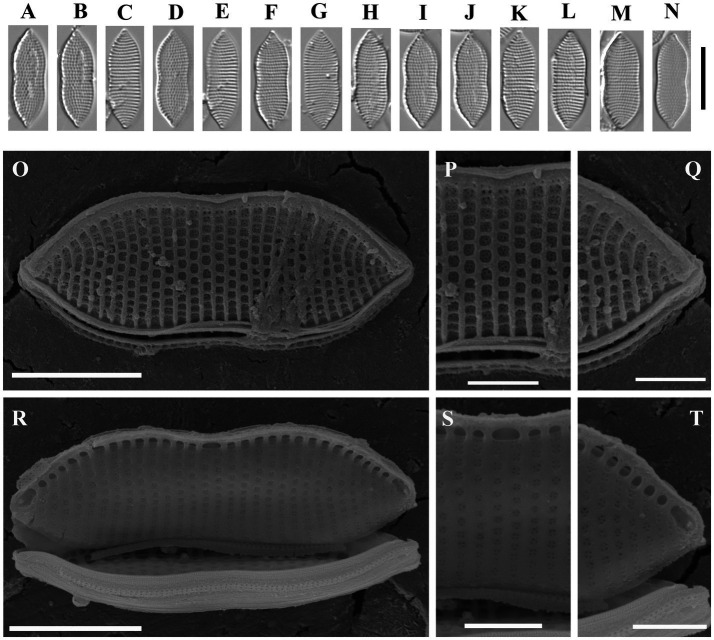
*Psammodictyon lanceolatum* sp. nov. **(A−N)** Size diminution series, LM; **(O−Q)** valve external view, SEM; **(O)** whole valve; **(P)** central part; **(Q)** valve apex; **(R−T)** valve internal view, SEM; **(R)** whole valve; **(S)** central part; **(T)** valve apex. Scale bars: **(A−N)** = 10 μm; **(O,R)** = 5 μm; **(P,Q,S,T)** = 2 μm.

Psammodictyon lanceolatum Kapustin, Kezlya and Kulikovskiy sp. nov. (Figure 6).

Description: LM: Valves lanceolate, slightly constricted in the middle, with cuneate ends, 15–16.5 μm long, 5–5.5 μm broad in the widest part, and 4.5–5 μm at the constriction. Keeled raphe system present on valve margin, with 15–19 distinct fibulae in 10 μm, the middle pair more distant from others. Striae almost parallel, 18–22 in 10 μm. SEM: Striae composed of loculate areolae with rectangular chambers. External raphe fissures accompanied by a ridge. Central raphe endings separated by a nodule. Distal raphe endings deflected to the mantle. Internally, areolae have 3–4 openings. Raphe terminates at apices in small helictoglossae.

Holotype: Permanent slide HD 09950, deposited at the K. A. Timiryazev Institute of Plant Physiology, Russian Academy of Sciences (HD), prepared from oxidized culture strain CBMCsvn866 isolated from sample NT-15 (40). Holotype illustrated in Figure 6D.

Isotype: Permanent slide 09950a, deposited at the Oceanographic Museum, Institute of Oceanography, Nha Trang, Viet Nam, with the accession number VMO: E 58220. Herbarium registration code VMO.

Type locality: Viet Nam, South China Sea, Thi Nai lagoon, Qui Nhon, plankton (N 13.827088, E 109.258570), *leg.* E. Kezlya and D. Kapustin, 27 July 2024.

Sequence data: Partial 18S rRNA gene sequence comprising V4 domain sequence (GenBank accession number PX596454), partial 18S rRNA gene sequence comprising V9-ITS1 domain sequence (GenBank accession number PX560130), and partial *rbcL* sequence (GenBank accession number PX607288) for the strain CBMCsvn866.

Reference strain: CBMCsvn866 deposited at the Culture and Barcode Collection of Microalgae and Cyanobacteria “AlgaBank” (CBMC), K. A. Timiryazev Institute of Plant Physiology, Russian Academy of Sciences and at the Microalgal Culture Collection of the Institute of Oceanography, Viet Nam.

Registration: https://phycobank.org/106052.

Etymology: The species epithet reflects the lanceolate valve shape.

**Figure 7:**
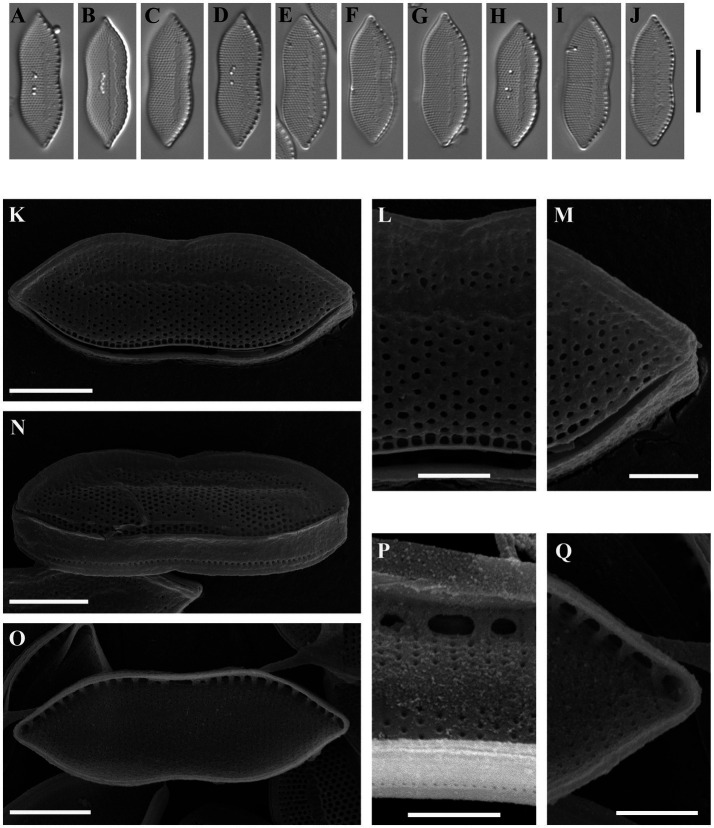
*Psammodictyon similis* sp. nov. **(A−J)** Size diminution series, LM; **(K−N)** valve external view, SEM; **(K)** whole valve; **(M)** central part; **(N)** valve apex; **(N)** girdle view; **(O−Q)** valve internal view, SEM; **(O)** whole valve; **(P)** central part (proximal raphe ends visible); **(Q)** valve apex. Scale bars: **(A−J)** = 10 μm; **(K,N,O)** = 5 μm; **(L,M,P,Q)** = 2 μm.

Psammodictyon similis Kapustin, Kezlya and Kulikovskiy sp. nov. (Figure 7).

Description: LM: Valves lanceolate, slightly constricted in the middle, with cuneate ends, and with hyaline sternum, 15–16.5 μm long, 5–5.5 μm broad in the widest part, and 4.5–5 μm at the constriction. Keeled raphe system present on valve margin, with 15–19 distinct fibulae in 10 μm, the middle pair more distant from others. Striae 18–22 in 10 μm, interrupted by a narrow hyaline area, areolae arranged in quincunx. SEM: Externally the proximal valve side (i.e., the part next to the raphe) slightly depressed and separated from the elevated distal side by a hyaline sternum. Striae decussate, composed of loculate areolae. External areolar openings round. External raphe fissures accompanied by a ridge. Central raphe endings separated by a nodule. Inner areolae openings round, slightly depressed. Raphe terminates at apices in small helictoglossae.

Holotype: Permanent slide HD 09818, deposited at the K. A. Timiryazev Institute of Plant Physiology, Russian Academy of Sciences (HD), prepared from oxidized culture strain CBMCsvn750 isolated from sample NT-3 (5). Holotype illustrated in Figure 7A.

Isotype: Permanent slide 09818a, deposited at the Oceanographic Museum, Institute of Oceanography, Nha Trang, Viet Nam, with the accession number VMO: E 58221. Herbarium registration code VMO.

Type locality: Viet Nam, South China Sea, Cua Tung, Quang Tri, plankton (N 17.022566, E 107.116105), *leg.* E. Kezlya and D. Kapustin, 23 July 2024.

Reference strain: CBMCsvn750 deposited at the Culture and Barcode Collection of Microalgae and Cyanobacteria “AlgaBank” (CBMC), K. A. Timiryazev Institute of Plant Physiology, Russian Academy of Sciences and at the Microalgal Culture Collection of Institute of Oceanography, Viet Nam.

Representative specimen. Strain CBMCsvn749 (slide no. 09817, sample no. NT-3 (5).

Sequence data: Partial 18S rRNA gene sequence comprising V4 domain sequence (GenBank accession number PX596456) and partial *rbcL* sequence (GenBank accession number PX607296) for the strain CBMCsvn750. Partial 18S rRNA gene sequence comprising V4 domain sequence (GenBank accession number PX596455) and partial *rbcL* sequence (GenBank accession number PX607297) for the strain CBMCsvn749.

Registration: https://phycobank.org/106053.

Etymology: The species epithet reflects the morphological similarity of the new species to the known *Psammodictyon* taxa.

**Figure 8:**
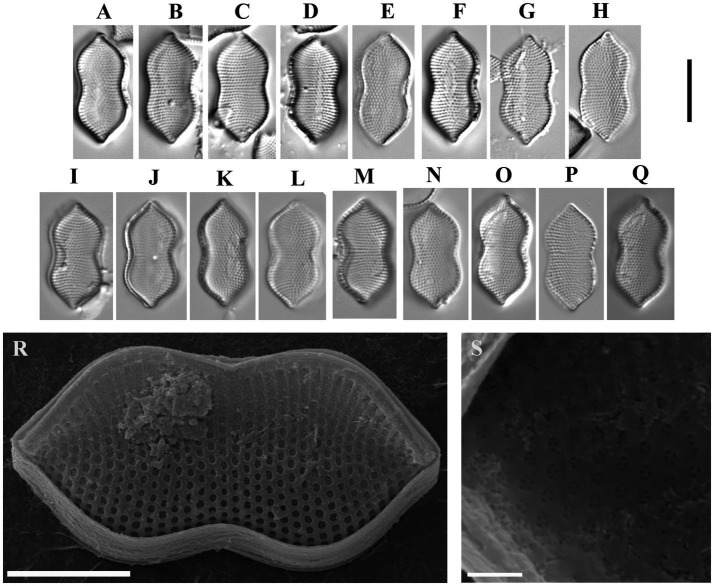
*Psammodictyon* cf. *constrictum*: **(A−Q)** size diminution series, LM; **(A−H)** strain CBMCsvn771; **(I−Q)** strain CBMCsvn773; **(R)** valve external view, strain CBMCsvn771, SEM; **(S)** close-up view of the valve near the apices internally, strain CBMCsvn771, SEM. Scale bars: **(A−Q)** = 10 μm; **(R)** = 5 μm, **(S)** = 1 μm.

Psammodictyon cf. constrictum (W. Gregory) D. G. Mann (Figure 8).

Description: LM: Valves panduriform, strongly constricted in the middle, with subrostrate ends, 17.3–18 μm long, 7.6–8.6 μm broad in the widest part, 6.3–7.3 μm at the constriction. Keeled raphe system present on valve margin, with 16 fibulae in 10 μm. Striae 23 in 10 μm. SEM: External locular openings round to elliptic. Internally, areolae have (2)3–4(5) openings.

Sequence data. Partial 18S rRNA gene sequence comprising V4 domain sequence (GenBank accession number PX596457) and partial *rbcL* sequence (GenBank accession number PX607298) for the strain CBMCsvn771. Partial 18S rRNA gene sequence comprising V4 domain sequence (GenBank accession number PX596459) and partial *rbcL* sequence (GenBank accession number PX607299) for the strain CBMCsvn773.

Representative specimens. Strain CBMCsvn771 (slide no. 09839) and strain CBMCsvn773 (slide no. 09841) from sample no. NT-19 (58).

### 3.2 Phylogenetic analysis

**Figure 9:**
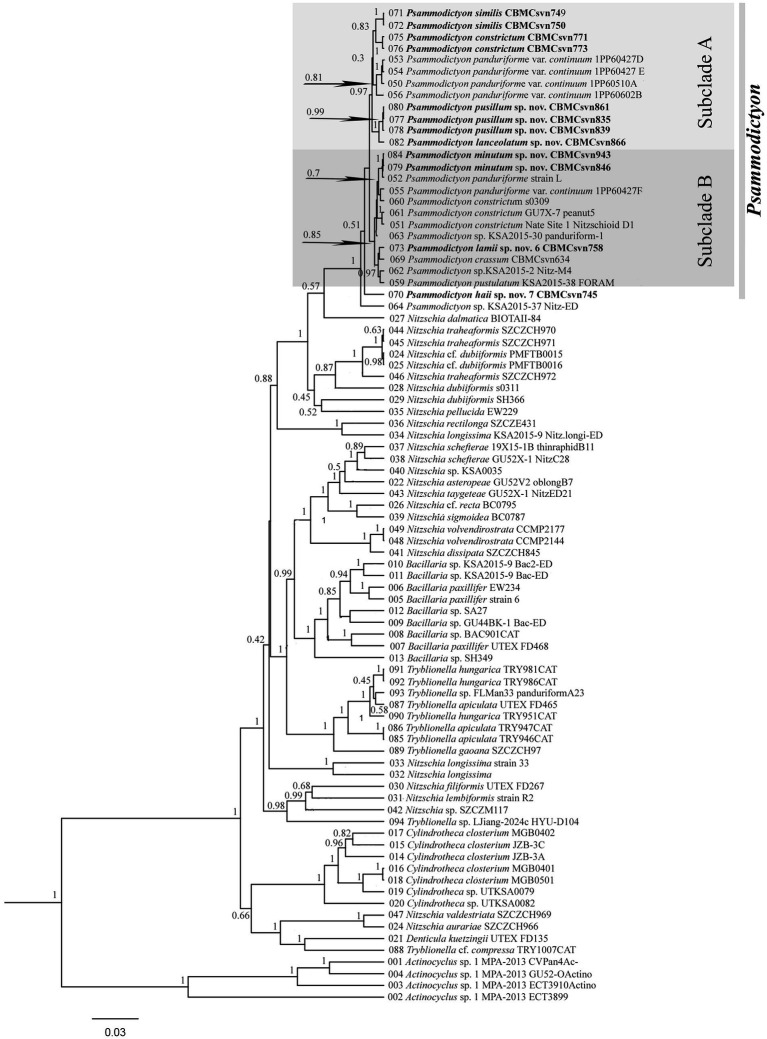
Phylogenetic position of the studied *Psammodictyon* strains (indicated in bold) based on Bayesian inference (BI) for the partial *rbc*L and SSU rRNA genes (87 sequences). The total alignment length is 1,614 characters. Posterior probabilities from BI (constructed in Beast) are presented at the nodes. Strain numbers are indicated for all sequences.

Phylogenetic analysis was performed on the nuclear region V4 of the 18S rRNA and the plastid *rbc*L. The final data set included 85 strains, of which 81 belong to the family Bacillariaceae (*Nitzschia*, *Tryblionella*, *Bacillaria*, *Cylindrotheca*, *Denticula*, *Psammodictyon*), with four strains of *Actinocyclus* Ehrenberg added as an outgroup. For the analysis of relationships within *Psammodictyon*, 13 strains of this genus from the GenBank and 12 strains studied here were included (Figure 9).

The phylogenetic analysis showed that the genera *Bacillaria* and *Cylindrotheca* form separate clades with maximum support (PP 1). *Nitzschia* strains are the most numerous in our dataset (n = 27) and were grouped into five clades that are scattered throughout the tree, showing polyphyly. Ten strains from the genus *Tryblionella* are included in the analysis, of which eight form the highest supported clade (PP 1), and two—*Tryblionella* sp. LJiang2024c HYU D104 and *T.* cf. *compressa* TRY1007CAT—with high supports (PP 0.98 and 1.0, respectively) belong to the clades with *Nitzschia* and *Nitzschia/Denticula*, respectively (see Figure 9).

*Psammodictyon* shows monophyly and forms a separate maximally supported clade (PP 1) near *Nitzschia dalmatica* and a clade that includes strains of *Nitzschia dubiiformis*, *N. tracheaformis*, and *N. pellucida*. Compared with other genera of Bacillariaceae, all *Psammodictyon* species are closely related to each other and are divided into two subclades (see Figure 9).

One subclade with a high statistical support (PP 0.97) consists of the strains of the species studied here, namely *P. similis* sp. nov. (CBMCsvn749, CBMCsvn750), *P. constrictum* (CBMCsvn771, CBMCsvn773), *P. pusillum* sp. nov. (CBMCsvn835, CBMCsvn839, CBMCsvn861), *P. lanceolatum* sp. nov. (CBMCsvn866), and strains of *P. panduriforme* var. *continuum* from GenBank. The second subclade (PP 0.85) includes strains of the new species *P. minutum* sp. nov. (CBMCsvn846, CBMCsvn943), *P. lamii* sp. nov. CBMCsvn758, the recently described *P. crassum* CBMCsvn634 (Kezlya et al., 2025b), and strains of *P. pustulatum* and *P. constrictum*. One more new species, *P. haii* sp. nov. CBMCsvn745, forms a separate lineage within the *Psammodictyon* clade.

The results of a pairwise similarity analysis based on *p*-distance showed that the sequences of the strains assigned to the same species based on morphological characteristics are completely identical across all studied regions (similarity = 100%) (Supplementary Tables S2–S7). For example, the obtained sequences for the V4 18S rRNA, *rbc*L, and V9-ITS1 regions for strains CBMCsvn861, CBMCsvn835, and CBMCsvn839 assigned to *P. pusillum* sp. nov., and for strains CBMCsvn846 and CBMCsvn943 assigned to *P. minutum* sp. nov., have 100% similarity.

The comparison of the V4 region of the 18S rRNA sequences (alignment length = 385 nt) revealed 100% similarity between eight strains belonging to two taxa—*P. constrictum* (CBMCsvn771, CBMCsvn773, strain s0309) and *P. panduriforme* var. *continuum* (Supplementary Table S2). These taxa also have high similarity (99.5–99.7%, which corresponds to differences of only 1–2 nt) with *P. similis* sp. nov. (strains CBMCsvn749, CBMCsvn750), *Psammodictyon* sp. KSA2015-2_Nitz-M4, *P. pustulatum* KSA2015-38, *P. lamii* sp. nov. CBMCsvn758, and *P. crassum* CBMCsvn634. In addition, there are no differences between strains belonging to *P. minutum* sp. nov. (CBMCsvn 846, CBMCsvn 943) and *P. panduriforme* strain L or between *P. pusillum* sp. nov. (CBMCsvn 835, CBMCsvn 839, CBMCsvn 861) and *P. lanceolatum* sp. nov. (CBMCsvn 866) in this region. Overall, for the V4 region of 18S rRNA, average pairwise sequence similarity values for *Psammodictyon* species range from 98% (*P.* sp. KSA2015-37_Nitz-ED) to 99.4% (*P. constrictum* CBMCsvn 771 and CBMCsvn773, *P. panduriforme* var. *continuum* (strains 1PP60427D, 1PP60427E, 1PP60510A), *P. constrictum* s0309) (Supplementary Table S2). Average intergeneric similarity values (*Psammodictyon/Tryblionella*) range from 93 to 94%.

For the *rbc*L region, two alignments were used for genetic distance analysis: the first included 25 strains of *Psammodictyon* with a length of 734 nt (Supplementary Table S3), and the second included 20 strains with a length of 1,071 nt (Supplementary Table S4). Both alignments showed similar results. Complete sequence similarity was observed between the strains belonging to the same species (*P. similis* (strains CBMCsvn771, CBMCsvn773), *P. constrictum* (strains CBMCsvn749, CBMCsvn750), *P. pusillum* sp. nov. (strains CBMCsvn835, CBMCsvn 839, CBMCsvn861), *P. minutum* sp. nov. (strains CBMCsvn943, CBMCsvn846), and between strains from GenBank (*P. constrictum* strains Nate Site1 and GU7X-7 peanut5)) (Supplementary Tables S3, S4). Average sequence similarities in pairwise comparisons within the genus range from 98%/97.9% (for *P. lanceolatum* sp. nov. CBMCsvn866) to 99.3% (*P. panduriforme* var. *continuum* (1PP60602B, 1PP60427F), *P. minutum* sp. nov. (CBMCsvn846, CBMCsvn943), *P. constrictum* (GU7X-7_peanut5, Nate Site 1), and *P. crassum* CBMCsvn634). The highest sequence similarities for the *rbc*L region (99.5–99.9%) were observed in both alignments in subclade B between *P. minutum* sp. nov./*P. panduriforme* var. *continuum* 1PP60427F/*P. constrictum*/*P.* sp. (strains KSA2015-30 panduriform-1, KSA2015-2_Nitz-M4) and *P. lamii* sp. nov., and between *P. crassum*/*P. constrictum* NateSite 1/*P.* sp. KSA2015-2_Nitz-M4 (Supplementary Tables S3, S4).

Sequence similarity values between genera do not exceed 91.7%.

### 3.2.1 Short barcode *rbc*L (p-distance)

An examination of the genetic distances between taxa using the *rbc*L region [~331 bp (379 bp with primers)], which is used in metagenomic studies of diatoms (Kezlya et al., 2023 and the references herein), showed results that are generally consistent with the same analysis performed on longer (734 and 1,071 nt) regions of this gene (Supplementary Table S5). However, the short barcode did not reveal any differences between the new species *P. pusillum* sp. nov. and *P. lanceolatum* sp. nov., while the similarity when comparing long regions does not exceed 99.4%. Also, short *rbc*L sequences completely coincided in *P. minutum* sp. nov. and *P. panduriforme* var. *continuum* 1PP60427F. It should be noted that despite the short length of the barcode region, in other cases, the percentage similarity values were close to the values obtained for long sections. That is, the short barcode region resolved almost all species. Average sequence similarity values for pairwise comparisons ranged from 97.7 to 98.8%. For the taxa of subclade B listed above, sequence similarity remained high (99.5–99.7%) (Supplementary Table S5).

#### 3.2.2 Barcode V9 and V9-ITS1 18S rRNA (p-distance)

We obtained sequences for the V9-ITS1 region for only nine of the 13 strains. For four strains, we were unable to amplify this region. There are currently no sequences of this region for *Psammodictyon* in the GenBank, so the dataset included sequences obtained in this study only (*n* = 9).

The analysis of sequence similarity in the V9 region of the 18S rRNA (alignment length 144 nt) showed that this region was conserved in the studied representatives of *Psammodictyon* (Supplementary Table S6). The sequences of all strains of *P. pusillum* sp. nov., *P. lanceolatum* sp. nov., and *P. minutum* sp. nov. are completely identical (similarity 100%); there were also no differences in the V9 region of the 18S rRNA between the new species *P. haii* sp. nov. and *P. lamii* sp. nov. The similarity values of the V9 18S rRNA region between *P. lamii* sp. nov., *P. crassum*, and *P. haii* sp. nov. range from 98.5 to 93.5%.

For region V9-ITS1 18S rRNA, the alignment length was 373 nt after eliminating gaps and missing data. This region turned out to be more variable compared with the short V9. Complete sequence similarity was noted only between strains assigned to the same species (*P. pusillum* sp. nov.—three strains, *P. minutum* sp. nov.—two strains; Supplementary Table S7 and Figure 9). Intraspecific variability in this region was not revealed. With the exception of *P. lanceolatum* sp. nov., which showed the maximum similarity values with strains of *P. pusillum* sp. nov. (99.7%), the sequence similarity for the remaining species does not exceed 94.1%. The minimum average similarity values (79.4–86.4%) were noted for *P. lamii* sp. nov., *P. crassum*, and *P. haii* sp. nov. (Supplementary Table S7).

### 3.3 Fatty acid profiles

Analysis of the biomass at the stationary phase of growth revealed the dominance of saturated 16:0 palmitic acid (within the range of 22.07–38% of total FA), monounsaturated 16:1n-7 palmitoleic acid (20.2–23.2%), and long-chain polyunsaturated 22:6(n-3) docosahexaenoic acid (12.57–22.69%) for all strains (Table 2). Similarly, all fatty acid profiles of investigated strains contained long-chain 20:5(n − 3) eicosapentaenoic acid (12.1–18.82%) and saturated 18:0 stearic acid (5.66–10.78%). A small amount of the long-chain saturated 22:0 behenic acid (1.82, 3.01, 1.33%) was detected in *P. similis* sp. nov. CBMCsvn749, *P. similis* sp. nov. CBMCsvn750, and *P. minutum* sp. nov. CBMCsvn846 profiles. In addition, a reduced concentration of the omega-6 adrenic acid (7.94, 2.12, 6.15, 6.5%) was recorded in *P. similis* CBMCsvn749, *P. lamii* sp. nov. CBMCsvn758, *P. pusillum* sp. nov. CBMCsvn839, and *P. minutum* sp. nov. CBMCsvn846.

## 4 Discussion

### 4.1 Comparison with similar taxa

Our study confirmed that the diversity of the genus *Psammodictyon* is underestimated, especially for the small-sized taxa. This study increased the number of known species in the genus up to 21 (Table 3), with six new species described. However, many more taxa remain undescribed, and some older *Nitzschia* taxa should be transferred to *Psammodictyon*.

**Table 3:** A list of known taxa of the genus *Psammodictyon*.

Taxon	Synonym(s)	Reference(s)
*Psammodictyon areolatum* (Hustedt) D. G. Mann	*Nitzschia areolata* Hustedt	Hustedt (1952)
*Psammodictyon bisculptum* (A. Mann) D. G. Mann	*Nitzschia bisculpta* A. Mann	Mann (1925)
*Psammodictyon bombiforme* (Grunow) D. G. Mann	*Nitzschia constricta* var. *bombiformis* Grunow	Cleve and Grunow (1880)
*Psammodictyon constrictum* (Gregory) D. G. Mann var. *constrictum*	*Tryblionella constricta* W. Gregory, *Nitzschia constricta* (W. Gregory) Grunow, nom. Illeg.	Gregory (1855)
*Psammodictyon constrictum* f. *parvum* (Grunow) Belegratis, Louvrou, and Economou-Amilli	*Nitzschia constricta* f. *parva* Grunow	Louvrou and Economou-Amilli (2012)
*Psammodictyon corpulentum* (N. I. Hendey) D. G. Mann	*Nitzschia corpulenta* N. I. Hendey	Hendey (1958)
*Psammodictyon crassum* Kapustin, Kezlya, and Kulikovskiy		Kezlya et al. (2025b)
*Psammodictyon ferox* (Hustedt) D. G. Mann	*Nitzschia ferox* Hustedt	Hustedt (1952)
*Psammodictyon inductum* (Hustedt) D. G. Mann	*Nitzschia inducta* Hustedt	Hustedt (1952)
*Psammodictyon mediterraneum* (Hustedt) D. G. Mann	*Nitzschia mediterranea* Hustedt 1921	Schmidt (1921)
*Psammodictyon molle* (Hustedt) D. G. Mann	*Nitzschia mollis* Hustedt	Hustedt (1952)
*Psammodictyon panduriforme* (Gregory) D. G. Mann var. *panduriforme*	*Nitzschia panduriformis* W. Gregory	Gregory (1857)
*Psammodictyon panduriforme* var. *continuum* (Grunow) P. Snoeijs	*Nitzschia panduriformis* var. *continua* Grunow	Cleve and Grunow (1880)
*Psammodictyon panduriforme* var. *delicatulum* (Grunow) M. Poulin	*Nitzschia panduriformis* var. *delicatula* Grunow 1880	Cleve and Grunow (1880) and Poulin et al. (1990)
*Psammodictyon panduriforme* var. *lata* (O. Witt) M.A. Harper	*Psammodictyon panduriforme* var. *lata* (O. Witt) Louvrou and Economou-Amilli, *Tryblionella lata* O. N. Witt, *Nitzschia panduriformis* var. *lata* (O. N. Witt) Cleve and Möller	Louvrou and Economou-Amilli (2012)
*Psammodictyon panduriforme* var. *peralbatum* (H. Peragallo and Peragallo) Louvrou and Economou-Amilli	*Nitzschia panduriformis* var. *peralbata* H. Peragallo and Peragallo	Louvrou and Economou-Amilli (2012)
*Psammodictyon pustulatum* (Voigt ex Meister) C.S. Lobban	*Nitzschia panduriformis* var. *pustulata* Voigt ex Meister	Meister (1937)
*Psammodictyon roridum* (M.H. Giffen) D.G. Mann	*Nitzschia rorida* M. H. Giffen	Giffen (1975)
*Psammodictyon rudum* (B.J. Cholnoky) D.G. Mann	*Nitzschia ruda* B. J. Cholnoky	Cholnoky (1968)
*Psammodictyon subconstrictum* (Grunow) Louvrou and Economou-Amilli	*Nitzschia constricta* var. *subconstricta* Grunow	Louvrou and Economou-Amilli (2012)
*Psammodictyon taihuense* Q. Yang, Q.-M. You and Q.-X. Wang	−	Yang et al. (2020)

The valve shape of *Psammodictyon lamii* is almost identical to *Nitzschia coarctata* var. *oceanica* Frenguelli (Frenguelli, 1928), but in the latter taxon, the hyaline area is shorter and located at the valve center, whereas in *P. lamii*, the hyaline area is located along the longitudinal axis. In addition, *N. coarctata* var. *oceanica* is slightly longer and wider. *Psammodictyon haii* is also similar to *Nitzschia coarctata* var. *oceanica* but differs in the lack of constriction and in having much smaller valves (17.5–19 × 7.5–8.5 *vs.* 30–39 × 13–16 μm, respectively).

Both *Psammodictyon minutum* and *P. pusillum* belong to the *P. constrictum* species complex. Unfortunately, Gregory (1855) did not provide any morphometric data, making adequate comparison impossible. Morphologically, *Psammodictyon minutum* and *P. pusillum* are virtually identical with overlapping morphometric data. However, in *P. minutum*, the striae are distinctly punctate. This distinction possibly results from differences in ultrastructure: in *P. pusillum*, the areolae are rectangular externally, whereas in *P. minutum*, the external areolar openings are round, except near the proximal valve margin, where the areolae remain rectangular. These differences probably have no taxonomic value and may be related to the silification process. Both taxa are similar to *P. constrictum apud* Lobban et al. (2012). We identified our strains CBMCsvn771 and CBMCsvn773 as *Psammodictyon* cf. *constrictum* because they appear close to the smaller specimen depicted by Gregory (1855). Gregory (1855) did not provide the size of this species but described it as “pretty little,” and all variants described by Cleve and Grunow (1880) have valve lengths larger than 60 μm, or, in the case of “*N. constricta* var. *genuina*,” 16–27 μm. The valves of *Psammodictyon* cf. *constrictum* (strains CBMCsvn771 and CBMCsvn773) in this study were 17.3–18 μm long, 7.6–8.6 μm broad at the widest part (6.3–7.3 μm at the constriction), with 23 striae in 10 μm and 16 fibulae in 10 μm.

The valves of *P. minutum* and *P. pusillum* are smaller (see Table 4), but the striae and the fibulae densities are comparable. Although the morphology and the phylogenetic placement of the type of *P. constrictum* remain unknown, we believe that the description of the new taxa, *P. minutum* and *P. pusillum*, is justified, as they originate from the South China Sea rather than from Europe.

**Table 4:** Morphological and morphometric comparison of the new *Psammodictyon* species with similar taxa.

Species	Valve shape	Valve apices	Hyaline area	Number of inner areolar openings	Valve length, μm	Valve width, μm	Valve width at the constriction, μm	Striae in 10 μm	Fibulae in 10 μm	Reference
*Psammodictyon lamii* sp. nov.	Broadly lanceolate	Subrostrate	+	1	29.5–30	11.5–13	11–12	20	9–10	This study
*Psammodictyon haii* sp. nov.	Elliptic	Cuneate to subrostrate	+	1	17.5–19	7.5–8.5	n/a	27–29	11–13	This study
*Psammodictyon lanceolatum* sp. nov.	Lanceolate	Cuneate	−	3–4	15–16.5	5–5.5	4.5–5	18–22	15–19	This study
*Psammodictyon minutum* sp. nov.	Panduriform	Cuneate to subrostrate	−	2–4	11–13.5	5.5–6.5	4.5–5.5	22	16	This study
*Psammodictyon pusillum* sp. nov.	Panduriform	Cuneate to subrostrate	−	3–4(5)	7–12	4.5–6	4.5–5	22–24	16–19	This study
*Psammodictyon* cf. *constrictum*	Panduriform	Subrostrate	−	(2)3–4	17.3–18	7.6–8.6	6.3–7.3	23	16	This study
*Psammodictyon similis*	Lanceolate	Subrostrate	+	1	20.2–22.0	6.9–7.5	6.1–6.8	24–26	8–12	This study
*Psammodictyon rudum*	Panduriform to linear	Rostrate	−	?	12–20	5.5–6.5	?	28–30	10–14	Cholnoky (1968)
*Psammodictyon roridum*	Panduriform to linear	Rostrate	+	?	23–32	9–10	8–9	24–27	10–12	Giffen (1975)
*Nitzschia coarctata* var. *oceanica*	Elliptic	Subrostrate	+	?	30–39	13–16	n/a	10–11	?	Frenguelli (1928)

In valve shape, *Psammodictyon lanceolatum* is similar to *Psammodictyon subconstrictum* (Grunow) Louvrou and Economou-Amilli (≡*Nitzschia panduriformis* var. *subconstricta* Grunow); however, the latter species has a hyaline sternum, which is absent in *P. lanceolatum*. In contrast to *Psammodictyon subconstrictum*, *P. lanceolatum* has 18–20 striae in 10 μm, whereas the former species has 11–12 striae in 10 μm (see Table 4).

Psammodictyon similis is morphologically related to *P. roridum*, from which it can be distinguished by its smaller size (20.2–22.0 × 6.9–7.5 μm (6.1–6.8 μm at the constriction) in *P. similis vs*. 23–32 × 9–10 μm (8–9 μm at the constriction) in *P. roridum*).

In *Psammodictyon*, two types of loculate areolae can be distinguished: (1) with rectangular chambers (e.g., *P. lanceolatum*, *P. minutum*, *P. pusillum*) and (2) with hexagonal chambers (e.g., *P. lamii*, *P. crassum*, *P. pustulatum* KSA2015-38 Foram). The chamber shape correlates with the number of inner areolar openings—one in hexagonal chambers and more than two (usually 3–4) in rectangular chambers. This feature may have a taxonomic value.

### 4.2 Phylogeny

In this study, the *Psammodictyon* dataset for the phylogenetic analysis was significantly expanded and includes a total of 25 strains. The phylogenetic tree shows that *Psammodictyon* species are closely grouped in the highest supported clade (PP 1) near *Nitzschia dubiiformis*, *N. tracheaformis*, *N. dalmatica*, and *N. pellucida* (Figure 9). The close relationship of *Psammodictyon* and the above-mentioned *Nitzschia* has been repeatedly demonstrated in previous studies, where the *Psammodictyon*/*Nitzschia* clade had high bootstrap support (99–100), regardless of the set of genetic markers chosen for analysis (Ashworth et al., 2017; Carballeira et al., 2017; Sabir et al., 2018; Mann et al., 2021; Mucko et al., 2021; Barkia et al., 2019). Compared with other representatives of Bacillariaceae, *Psammodictyon* has low interspecific genetic distances.

In general, low interspecific distances (0.1–0.5% or 1–5 bp) for the V4 18S rRNA and *rbc*L marker regions have been repeatedly observed in closely related diatom species, for example, in closely related species of *Sellaphora* Mereschkowsky (Hamsher et al., 2011; Vanormelingen et al., 2013), *Pinnularia*, (Poulíčková et al., 2018; Kollár et al., 2019; Kollár et al., 2021; Pinseel et al., 2019), *Achnanthidium minutissimum* (Kützing) Czarnecki species complex (Pinseel et al., 2017), and *Humidophila* (Lange-Bertalot et Werum) R. L. Lowe et al. (Kezlya et al., 2025a). *Psammodictyon* studied here demonstrates a similar case.

The results of pairwise sequence comparison show that differences between species in the V4 region of the 18S rRNA can be only 0.3–0.5% (or 1–2 bp), for example, between *P. similis* sp. nov., *P. constrictum*, *P. pustulatum, P. lamii* sp. nov., and *P. crassum* (Supplementary Table S2).

The average sequence similarity values for the V4 region of the 18S rRNA vary from 98 to 99.4%. It is important to note that this region does not separate all species. In pairwise comparison, 100% sequence similarity was noted for the pairs *P. constrictum*/*P. panduriforme* var. *continuum*, *P. pusillum* sp. nov./*P. lanceolatum* sp. nov., and *P. minutum* sp. nov./*P. panduriforme*.

The *rbc*L gene allows separation of all *Psammodictyon* species regardless of the alignment length (734 or 1,071 bp), and no cases of complete sequence coincidence between different species were noted. Sequence differences between species generally exceed 1%, with average similarity values between species varying from 98 to 99.3% (Supplementary Tables S3, S4). The highest similarity (99.5–99.7%) was found between the group *P. minutum* sp. nov./*P. panduriforme* var. *continuum* 1PP60427F*/P. constrictum* s0309/*P. lamii* sp. nov. and the pair *P. crassum*/*P. constrictum* (GU7X-7_peanut5, NateSite 1). The latter are better differentiated by the V4 region of the 18S rRNA (98.7–98.9%). This group, except for *P. lamii* sp. nov. described here, includes small-celled species (Table 4) isolated from the South China Sea (*P. minutum* sp. nov. described here) and from the Black Sea, Russia (*P. panduriforme* var. *continuum* 1PP60427F; unpublished, location data from GenBank), while *P. constrictum* s0309 has no data about the isolation site. Their V4 region of the 18S rRNA also shows high similarity 99.5%, indicating a close relationship. *P. lamii* sp. nov. is separated from this group by the V4 18S rRNA (similarity 98.7%).

The small-celled species *P. pusillum* sp. nov. and *P. lanceolatum* sp. nov. are grouped into one branch (Figure 9). They differ very slightly in the *rbc*L gene (similarity 99.2–99.4%) and are completely identical in the marker regions V4, V9, and V9-ITS1 of the 18S rRNA (Supplementary Tables S2, S6, S7) and in the short barcode region of the *rbc*L gene (Supplementary Table S5). Nevertheless, both species clearly differ in valve shape and size (Table 4).

Our dataset contains five strains identified as *P. constrictum*: three from the GenBank (s0309, GU7X-7_peanut5, NateSite 1) and two strains studied here (CBMCsvn771, CBMCsvn773). Strains from GenBank are grouped in subclade B, whereas our strains form a separate branch in subclade A near *P. similis* (Figure 9). Sequence similarity between these groups for the *rbc*L gene does not exceed 99.2% (Supplementary Tables S3–S5); for the V4 region of 18S rRNA, 100% similarity was observed between the strains studied here and s0309, and 99.5% with the others. We identified our strains CBMCsvn771 and CBMCsvn773 as *P. constrictum* because we believe that the valve outline of our specimens is the closest to Gregory’s figure (Gregory, 1855, Figure 13). Dr. Matt Ashworth (UTEX Culture Collection of Algae) provided SEM micrographs of *P. constrictum* GU7X-7_peanut5 and *P. constrictum* NateSite 1. Morphologically, both strains are similar to each other and to our strains belonging to *P.* cf. *constrictum*, *P. pusillum*, and *P. minutum*. However, *P. constrictum* GU7X-7_peanut5 is larger than *P. constrictum* NateSite 1 (17.8–18.1 × 7.8–8.5 μm (7.6–8.0 μm at the constriction) vs. 11 × 5.2 μm (5.4 μm at the constriction) respectively). In addition, both strains have 3–4 internal areolar openings. Thus, *P. constrictum* represents a complex of cryptic species that requires further revision.

Owing to the high similarity of sequences among different species for the *rbc*L gene, especially in subclade B, we hypothesized that the short barcode region of the *rbc*L gene (331 bp), which is used for diatom metabarcoding (Vasselon et al., 2017; Kelly et al., 2018; Pérez-Burillo et al., 2022; Kezlya et al., 2023, and references herein), would not be able to distinguish the species. Our *in silico* validation showed that the short barcode region of *rbc*L can distinguish almost all species (except for the pairs *P. pusillum* sp. nov./*P. lanceolatum* sp. nov. and *P. minutum* sp. nov./*P. panduriforme* var. *continuum* 1PP60427F). The sequence similarity values are close to those obtained for the full-length gene.

Currently, the V9 region of the 18S rRNA is frequently used for metabarcoding of eukaryotic communities (van der Loos and Nijland, 2021; Kezlya et al., 2023), and it has been chosen to amplify eukaryotes in the global project “The Earth Microbiome Project” (EMP; https://earthmicrobiome.org/ (accessed on 25 August 2025)). The V4 region of the 18S rRNA is characterized as more variable when assessing the eukaryotic communities (Stoeck et al., 2010; Tanabe et al., 2016; Choi and Park, 2020; Tragin et al., 2018); however, a smaller reference dataset is available for V9 in diatoms (Piredda et al., 2017). The 18S rRNA gene sequences of *Psammodictyon* strains included in our analysis did not contain this region, so the comparison was performed only between the strains obtained in this study. Considering that only three of six species included in the analysis can be distinguished by the V9 region of the 18S rRNA, it can be concluded that this region is conservative in *Psammodictyon* and not effective for species separation.

The longer V9-ITS1 barcode region, which is also used for metabarcoding, proved to be much more effective (Kezlya et al., 2023 and the references herein). Despite the known issues of intragenomic variability in diatom ITS regions (Behnke et al., 2004; Evans et al., 2007; Kermarrec et al., 2013), no intraspecific variability was observed in our small sample of *P. pusillum* sp. nov and *P. minutum* sp. nov strains. Considering that all species were differentiated by this region, the sequence similarity level was very low compared to the V4 region of the 18S rRNA and *rbc*L markers and ranged from 78.5 to 94.1%, except for *P. pusillum* sp. nov./*P. lanceolatum* sp. nov. (similarity 99.7%).

### 4.3 Fatty acid content in Bacillariales

Сomparable values were obtained for marine *Cylindrotheca* strains, which accumulate a high percentage of PUFAs during the exponential phase (29.5–42.9%), especially 20:5 (*n*-3) and 20:4 (*n*-6) (Li et al., 2014). The dominant FAs in marine diatoms *Nitzschia closterium* CS-5 and *Cylindrotheca fusiformis* CS-13 were also reported as saturated 16:0 palmitic acid (7.2, 20%), monounsaturated 16:1n-7 palmitoleic acid (22.8, 19.7%), and long-chain 20:5(*n*−3) eicosapentaenoic acid (24.2, 20.3%) (Dunstan et al., 1993). Despite the fact that marine diatoms are considered promising sources of PUFA, marine strains *P. constrictum* MACC 34 and *P. panduriforme* MACC 35 are characterized by monounsaturated 16:1n-7 palmitoleic acid (15.26, 17.77%) and SFAs. In *P. constrictum* MACC 34, the dominant ones are 18:0 stearic acid (15.83%), 22:0 behenic acid (16.98%), and 24:0 lignoceric acid (22.97%), while in *P. panduriforme* MACC 35, the dominant FA is 18:0 stearic acid (58.37%) (Krishnaswami et al., 2024).

The study of FA composition in the examined *Psammodictyon* strains is particularly important given the general lack of biochemical studies on diatoms from Viet Nam and the near absence of research focused on this genus.

It is difficult to determine why the results obtained in this study differ so markedly from the literature data, as the available dataset for this genus remains limited. Our results suggest that genus *Psammodictyon*, like most diatoms, accumulates high levels of polyunsaturated fatty acids (29.44–42.62%, with a maximum of 42.62% in *P. minutum* sp. nov. CBMCsvn846) and monounsaturated fatty acids (20.2–23.2%, with a maximum of 23.2% in *P. minutum* sp. nov. CBMCsvn846). Based on similar studies, it can be concluded that in marine diatoms, during the stationary growth phase, the dominant FAs are eicosapentaenoic, docosahexaenoic, and palmitoleic acids (Zhukova and Aizdaicher, 1995; Ying et al., 2000; Mao et al., 2020). It might be reasonable, therefore, to consider *Psammodictyon* strains as potential producers of long-chain omega-3 polyunsaturated fatty acids used in various fields such as medicine (Pereira et al., 2012; Dhanker et al., 2023), aquaculture (Yaakob et al., 2014), cosmetology (De Luca et al., 2021), and biotechnology (Barta et al., 2021). The role of diatoms in aquaculture with such characteristics is also beyond doubt, as they are the primary source of nutrients for secondary consumers in the food chain (Dhanker et al., 2024). Furthermore, diatoms rich in polyunsaturated fatty acids are an ideal option for biofuel production, given that—with the proper approach to cultivation and using the necessary equipment—they offer the lowest production costs with the highest yield of the final product (Dhanker et al., 2024). It is also worth noting that the use of algae for biofuel is essentially waste-free, as algae, in addition to PUFA, store proteins, antioxidants, vitamins, and other chemicals that can be utilized by humans as dietary supplements, agricultural fertilizers, or in medicine (Dhanker et al., 2022; Pereira et al., 2012).

## 5 Conclusion

Our phylogenetic analysis showed that *Psammodictyon* represents a closely related monophyletic group. Interspecific distances for the V4 region of 18S rRNA and *rbc*L were often no more than 1% (sometimes only 0.3–0.5%), and overall sequence similarity in the studied sample did not exceed 97.3%, with average values of 98–99.4%. Assessment of the resolving power of short barcodes [V4, V9, V9-ITS1 regions of 18S rRNA, short *rbc*L (331 bp)] for species separation showed that V9-ITS1 was the most variable (for five of six studied species, the similarity did not exceed 94.1%). The V4 and V9 regions of 18S rDNA failed to distinguish almost half of the species included in this analysis. The short *rbc*L fragment, except for two species pairs, effectively differentiated all *Psammodictyon* taxa, yielding sequence similarity values comparable to those obtained for the full-length gene. These findings are important to consider, for instance, when analyzing metagenomic data and when selecting genetic markers and thresholds for species assignment.

The fatty acid content in the cells of the studied *Psammodictyon* strains revealed increased proportions of palmitoleic, eicosapentaenoic, and docosahexaenoic acids, which are typical for marine diatoms. Therefore, these strains may be considered promising for cultivation as potential sources of long-chain polyunsaturated and monounsaturated fatty acids relevant to biotechnological applications.

## Data Availability

The datasets presented in this study can be found in online repositories. The names of the repository/repositories and accession number(s) can be found in the article/Supplementary material. The sequencing data for the strains CBMCsvn745, CBMCsvn749, CBMCsvn750, CBMCsvn758 CBMCsvn771, CBMCsvn773, CBMCsvn835, CBMCsvn 839, CBMCsvn846, CBMCsvn861, CBMCsvn866, CBMCsvn943 are available from the authors upon request.
