# Supplementary material for: Comprehensive analysis of the diatom genus Psammodictyon from Viet Nam: new species, molecular data, and fatty acid content
Source: Front Microbiol. 2025 Dec 12;16:1701605. doi: 10.3389/fmicb.2025.1701605 (PMC12742474; doi:10.3389/fmicb.2025.1701605)
Supplement: Supplementary file 2 [file Table_2.DOCX]

| **Supplementary table S1.** Taxa and DNA sequence data used in phylogenetic analysis | | | |  |
| --- | --- | --- | --- | --- |
| **no.** | **Taxa** | **Strain** | **The GenBank accession no** | |
|  |  |  | ***rbc*L** | **18S rRNA** |
| 1 | *Actinocyclus* sp. 1 | MPA-2013 ECT3910Actino | KC309594 | KC309521 |
| 2 | *Actinocyclus* sp. 1 | MPA-2013 CVPan4Actino | KC309597 | KC309524 |
| 3 | *Actinocyclus* sp. 1 | MPA-2013 ECT3899tinydrum | KC309595 | KC309522 |
| 4 | *Actinocyclus* sp. 1 | MPA-2013GU52-OActino | KC309596 | KC309523 |
| 5 | *Bacillaria paxillifer* | strain 6 | KJ671796 | KJ671688 |
| 6 | *Bacillaria paxillifer* | EW234 | KY320315 | KY320376 |
| 7 | *Bacillaria paxillifer* | UTEX FD468 | HQ912491 | HQ912627 |
| 8 | *Bacillaria* sp. | BAC901CAT | MN734075 | MN750450 |
| 9 | *Bacillaria* sp. | GU44BK-1 Bac-ED | MH064066 | MH063448 |
| 10 | *Bacillaria* sp. | KSA2015-9 Bac2-ED | MH064069 | MH063451 |
| 11 | *Bacillaria* sp. | KSA2015-9 Bac-ED | MH064068 | MH063450 |
| 12 | *Bacillaria* sp. | SA27 | MH064067 | MH063449 |
| 13 | *Bacillaria sp.* | SH349 | KY320316 | KY320377 |
| 14 | *Cylindrotheca closterium* | JZB-3A | DQ143046 | DQ178392 |
| 15 | *Cylindrotheca closterium* | JZB-3C | DQ143047 | DQ178393 |
| 16 | *Cylindrotheca closterium* | MGB0401 | AY866416 | AY866417 |
| 17 | *Cylindrotheca closterium* | MGB0402 | AY866415 | AY866418 |
| 18 | *Cylindrotheca closterium* | MGB0501 | DQ019445 | DQ019446 |
| 19 | *Cylindrotheca* sp. | UTKSA0079 | KX981826 | KX981848 |
| 20 | *Cylindrotheca* sp. | UTKSA0082 | KX981827 | KX981847 |
| 21 | *Denticula kuetzingii* | UTEX FD135 | HQ912474 | HQ912610 |
| 22 | *Nitzschia asteropeae* | GU52V2 oblongB7 | MW324605 | MW327180 |
| 23 | *Nitzschia aurariae* | SZCZCH966 | KT943663 | KT943639 |
| 24 | *Nitzschia* cf. *dubiiformis* | PMFTB0015 | PP374839 | PP393120 |
| 25 | *Nitzschia* cf. *dubiiformis* | PMFTB0016 | PP374840 | PP393121 |
| 26 | *Nitzschia* cf. *recta* | BC0795 | MN718791 | MN750424 |
| 27 | *Nitzschia dalmatica* | BIOTAII-84 | MH687908 | MH734172 |
| 28 | *Nitzschia dubiiformis* | s0311 | AB430696 | AB430616 |
| 29 | *Nitzschia dubiiformis* | SH366 | KY320321 | KY320382 |
| 30 | *Nitzschia filiformis* | UTEX FD267 | HQ912453 | HQ912589 |
| 31 | *Nitzschia lembiformis* | strain R2 | HE802701 | MN750493 |
| 32 | *Nitzschia longissima* |  | KJ671808 | AY881968 |
| 33 | *Nitzschia longissima* | strain 33 | KJ671808 | KJ671700 |
| 34 | *Nitzschia longissima* | KSA2015-9 Nitz.longi-ED | MH064112 | MH063481 |
| 35 | *Nitzschia pellucida* | EW229 | KY320328 | KY320389 |
| 36 | *Nitzschia rectilonga* | SZCZE431 | MN920679 | MN943999 |
| 37 | *Nitzschia schefterae* | 19X15-1B thinraphidB11 | MN920679 | MW327181 |
| 38 | *Nitzschia schefterae* | GU52X-1 NitzC28 | MW324607 | MW327182 |
| 39 | *Nitzschia sigmoidea* | BC0787 | MN718790 | MN750423 |
| 40 | *Nitzschia* sp. | KSA0035 | KU179116 | KU179128 |
| 41 | *Nitzschia dissipata* | SZCZCH845 | KT943665 | KT943641 |
| 42 | *Nitzschia* sp. | SZCZM117 | KU179115 | KU179129 |
| 43 | *Nitzschia taygeteae* | GU52X-1 NitzED21 | MW324615 | MW327189 |
| 44 | *Nitzschia traheaformis* | SZCZCH970 | KT943666 | KT943642 |
| 45 | *Nitzschia traheaformis* | SZCZCH971 | KT943667 | KT943643 |
| 46 | *Nitzschia traheaformis* | SZCZCH972 | KT943668 | KT943644 |
| 47 | *Nitzschia valdestriata* | SZCZCH969 | KT943664 | KT943640 |
| 48 | *Nitzschia volvendirostrata* | CCMP2144 | MW324612 | MW327186 |
| 49 | *Nitzschia volvendirostrata* | CCMP2177 | MW324613 | MW327187 |
| 50 | *Psammodictyon panduriforme* var. *continuum* | 1PP60510A | MH390345 | MG983989. |
| 51 | *Psammodictyon constrictum* | Nate Site 1 Nitzschioid D1 | MH040278 | MH040329 |
| 52 | *Psammodictyon panduriforme* | strain L | KJ671809 | KJ671701 |
| 53 | *Psammodictyon panduriforme* var. *continuum* | 1PP60427D | MH390342 | MG983987 |
| 54 | *Psammodictyon panduriforme* var. *continuum* | 1PP60427E | MH390343 | MG983988 |
| 55 | *Psammodictyon panduriforme* var. *continuum* | 1PP60427F | MH390344 | MG992472 |
| 56 | *Psammodictyon panduriforme* var. *continuum* | 1PP60602B | MH390348 | MG983991 |
| 57 | *Psammodictyon pustulatum* | KSA2015-38 FORAM pandur-FO1 | MH064134 | MH063502 |
| 58 | *Psammodictyon constrictum* | s0309 | AB430697 | AB430617 |
| 59 | *Psammodictyon constrictum* | GU7X-7 peanut5 | KX981830 | KX981851 |
| 60 | *Psammodictyon* sp. | KSA2015-2 Nitz-M4 | MH064133 | MH063501 |
| 61 | *Psammodictyon* sp. | KSA2015-30 panduriform-1 | MH064131 | MH063499 |
| 62 | *Psammodictyon* sp | KSA2015-37 Nitz-ED | MH064132 | MH063500 |
| 63 | ***Psammodictyon similis* sp.nov.** | **CBMCsvn749** |  |  |
| 64 | ***Psammodictyon similis* sp.nov.** | **CBMCsvn750** |  |  |
| 65 | ***Psammodictyon constrictum*** | **CBMCsvn771** |  |  |
| 66 | ***Psammodictyon* *constrictum*** | **CBMCsvn773** |  |  |
| 67 | ***Psammodictyon pusillum* sp. nov.** | **CBMCsvn861** |  |  |
| 68 | ***Psammodictyon pusillum* sp. nov.** | **CBMCsvn835** |  |  |
| 69 | ***Psammodictyon pusillum* sp. nov.** | **CBMCsvn839** |  |  |
| 70 | ***Psammodictyon* *lanceolatum* sp. nov.** | **CBMCsvn86** |  |  |
| 71 | ***Psammodictyon minutum* sp. nov.** | **CBMCsvn943** |  |  |
| 72 | ***Psammodictyon minutum* sp. nov.** | **CBMCsvn846** |  |  |
| 73 | ***Psammodictyon lamii* sp. nov.** | **CBMCsvn75** |  |  |
| 74 | ***Psammodictyon haii* sp. nov.** | **CBMCsvn745** |  |  |
| 75 | *Psammodictyon crassum* | CBMCsvn634 | PX210930 | PX208944 |
| 76 | *Tryblionella apiculata* | TRY946CAT | MN734089 | MN750507 |
| 77 | *Tryblionella apiculata* | TRY947CAT | MN734090 | MN750508 |
| 78 | *Tryblionella apiculata* | UTEX FD465 | HQ912464 | HQ912600 |
| 79 | *Tryblionella* cf. *compressa* | TRY1007CAT | MN734088 | MN750506 |
| 80 | *Tryblionella gaoana* | SZCZCH97 | KT943683 | KT943644 |
| 81 | *Tryblionella hungarica* | TRY951CAT | MN734091 | MN750509 |
| 82 | *Tryblionella hungarica* | TRY981CAT | MN734092 | MN750510 |
| 83 | *Tryblionella hungarica* | TRY986CAT | MN734093 | MN750511 |
| 84 | *Tryblionella* sp. | FLMan33 panduriformA23 | MW324632 | MH017645 |
| 85 | *Tryblionella* sp. | LJiang-2024c HYU-D104 | PP883546 | PP839784 |
